# Aqua­[4-chloro-2-(2-pyridylmethyl­imino­meth­yl)phenolato]copper(II) nitrate monohydrate

**DOI:** 10.1107/S1600536809052350

**Published:** 2009-12-12

**Authors:** Qing Liang, Xiaodan Chen, Huaihong Zhang, Zhihong Zou

**Affiliations:** a Ordered Matter Science Research Center, College of Chemistry and Chemical Engineering, Southeast University, Nanjing 210096, People’s Republic of China

## Abstract

In the title mononuclear complex, [Cu(C_13_H_10_ClN_2_O)(H_2_O)]­NO_3_·H_2_O, the Cu^II^ atom is four-coordinated by two N atoms and one O atom of the tridentate Schiff base ligand and one O atom from the coordinated water mol­ecule in a slightly distorted square-planar configuration. The nitrate ion inter­acts with the copper center [Cu1⋯O3 = 2.579 (4) Å]. In the crystal, the cations, anions and water mol­ecules are linked by O—H⋯O and O—H⋯N hydrogen bonds.

## Related literature

For the role of copper proteins in fundamental biological processes, see: Arnesano *et al.* (2004 ▶). For the chemistry of copper compounds, see: Bosnich (1968 ▶); Costes *et al.* (1995 ▶); Downing & Urbach (1969 ▶); Ganeshpure *et al.* (1996 ▶). For related structures, see: Sun *et al.* (2005 ▶); You *et al.* (2004 ▶). 
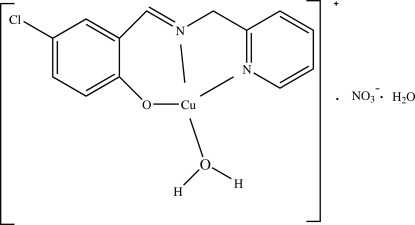


## Experimental

### 

#### Crystal data

[Cu(C_13_H_10_ClN_2_O)(H_2_O)]NO_3_·H_2_O
*M* *_r_* = 407.26
Triclinic, 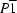

*a* = 7.892 (2) Å
*b* = 8.9741 (12) Å
*c* = 11.8929 (15) Å
α = 106.841 (2)°
β = 102.198 (1)°
γ = 92.897 (1)°
*V* = 782.3 (2) Å^3^
*Z* = 2
Mo *K*α radiation
μ = 1.60 mm^−1^
*T* = 298 K
0.47 × 0.41 × 0.30 mm

#### Data collection

Rigaku SCXmini diffractometer
Absorption correction: multi-scan (*CrystalClear*; Rigaku, 2005 ▶) *T*_min_ = 0.520, *T*_max_ = 0.645
4114 measured reflections
2714 independent reflections
2280 reflections with *I* > 2σ(*I*)
*R*_int_ = 0.016

#### Refinement

*R*[*F* ^2^ > 2σ(*F* ^2^)] = 0.030
*wR*(*F* ^2^) = 0.074
*S* = 1.06
2714 reflections
218 parameters
1 restraint
H-atom parameters constrained
Δρ_max_ = 0.40 e Å^−3^
Δρ_min_ = −0.39 e Å^−3^

### 

Data collection: *CrystalClear* (Rigaku, 2005 ▶); cell refinement: *CrystalClear*; data reduction: *CrystalClear*; program(s) used to solve structure: *SHELXS97* (Sheldrick, 2008 ▶); program(s) used to refine structure: *SHELXL97* (Sheldrick, 2008 ▶); molecular graphics: *SHELXTL* (Sheldrick, 2008 ▶); software used to prepare material for publication: *SHELXTL*.

## Supplementary Material

Crystal structure: contains datablocks I, global. DOI: 10.1107/S1600536809052350/zq2021sup1.cif

Structure factors: contains datablocks I. DOI: 10.1107/S1600536809052350/zq2021Isup2.hkl

Additional supplementary materials:  crystallographic information; 3D view; checkCIF report

## Figures and Tables

**Table:** 

Cu1—O1	1.889 (2)
Cu1—N1	1.936 (3)
Cu1—O2	1.975 (2)
Cu1—N2	1.982 (3)

**Table:** 

O1—Cu1—N1	93.94 (10)
O1—Cu1—O2	88.85 (9)
N1—Cu1—O2	171.60 (10)
O1—Cu1—N2	176.81 (10)
N1—Cu1—N2	82.98 (11)
O2—Cu1—N2	94.32 (10)

**Table 2:** Hydrogen-bond geometry (Å, °)

*D*—H⋯*A*	*D*—H	H⋯*A*	*D*⋯*A*	*D*—H⋯*A*
O2—H2a⋯O5	0.85	1.83	2.676 (4)	173
O2—H2a⋯N3	0.85	2.52	3.253 (4)	146
O2—H2a⋯O3	0.85	2.57	3.052 (4)	117
O2—H2b⋯O6^i^	0.85	1.81	2.657 (4)	174
O6—H6a⋯O1^ii^	0.85	2.08	2.915 (3)	166
O6—H6b⋯O4	0.85	1.93	2.782 (5)	177

Symmetry codes: (i) 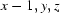; (ii) 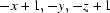.

**Fig. 1.**
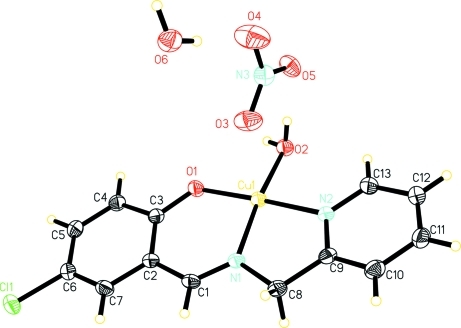
The molecular structure of the title molecule, with the atom-numbering scheme. Displacement ellipsoids are drawn at the 30% probability level.

**Fig. 2.**
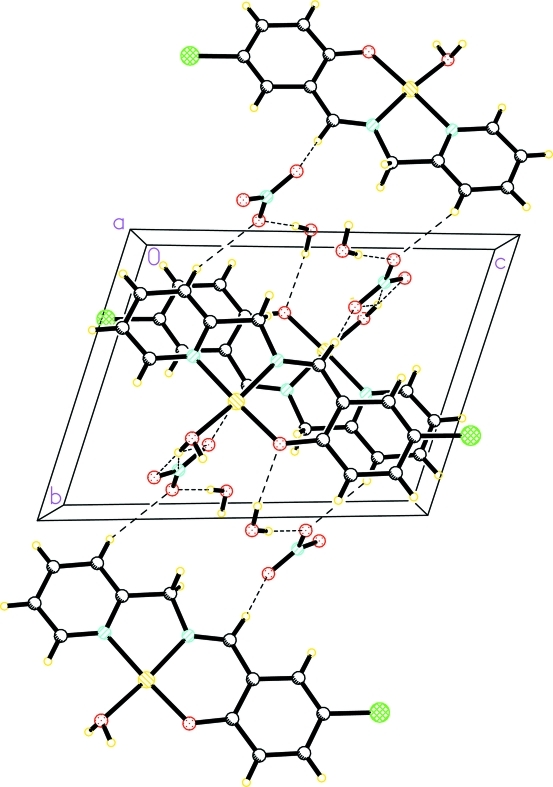
Crystal packing of the compound (I). Hydrogen bonds are shown as dashed lines.

## supplementary crystallographic information

### Comment

Metals ions are vital for living organisms because they are involved in many fundamental biological processes, *e.g.* copper proteins known to be involved in a crucial role, such as respiration, iron transport, oxidative stress protection, blood clotting and pigmentation (Arnesano *et al.*, 2004). The study of copper compounds is of great interest in various aspects of chemistry (Downing & Urbach, 1969; Ganeshpure *et al.*, 1996; Bosnich, 1968; Costes *et al.*, 1995). The molecular structure of (I) is illustrated in Fig. 1, and selected bond distances and angles are given in Table 1. The Cu^II^ atom is four- coordinated by two nitrogen atoms and one oxygen atom of the tridentate Schiff base ligand, and one oxygen atom from the coordinated water molecule, forming a slightly distorted square-planar coordination configuration. The four coordinating atoms around the Cu centre are approximately coplanar. The Cu1—N2 bond [1.982 (2) Å; Table 1] is a little longer than the value [1.977 (4) Å] observed in a similar copper(II) complex (Sun *et al.*, 2005). The Cu1—N1 bond length [1.936 (2) Å] is comparable with the corresponding value [1.934 (4) Å] observed in the same complex mentioned above (Sun *et al.*, 2005). The Cu1—O1 bond length is 1.889 (18) Å. The nitrate ion is in interaction with the copper center [Cu1···O3 = 2.579 (4) Å]. The bond angles around the Cu^II^ centre show some deviations from ideal square-planar geometry. The Schiff base ligands from adjacent molecules are almost parallel due to by π-π interactions leading to the formation of two-dimensional parallel layers (Fig.2). The cations, anions and solvent water molecules are linked by O-H···O hydrogen bonds.

### Experimental

2-Aminomethylpyridine (0.1 mmol, 10.8 mg) and 5-chloro-salicylaldehyde (0.1 mmol, 15.6 mg) were dissolved in methanol (10 ml). The mixture was stirred for 1 h to give a clear yellow solution. To this solution was added a water solution (10 ml) of Cu(NO_3_)2.3H_2_O (0.1 mmol, 24.2 mg), with stirring. The mixture was stirred for 10 min to give a deep green solution, which was allowed to evaporate slowly in the open at room temperature. After 5 days, deep blue block-shaped crystals suitable for an X-ray diffraction study were formed at the bottom of the vessel.

### Refinement

The hydrogen atoms bound to carbon atoms were placed in geometrical positions and refined using a riding model, with C—H = 0.94 Å and *U*_iso_(H) =1.2*U*_eq_(C). The hydrogens of the water molecules were located in Fourier difference maps and refined with a distance restraint of 0.85 Å.

### Crystal data

**Table:**

[Cu(C_13_H_10_ClN_2_O)(H_2_O)]NO_3_·H_2_O	*Z* = 2
*M**_r_* = 407.26	*F*(000) = 414
Triclinic, *P*1	*D*_x_ = 1.729 Mg m^−^^3^
Hall symbol: -P 1	Mo *K*α radiation, λ = 0.71073 Å
*a* = 7.892 (2) Å	Cell parameters from 13380 reflections
*b* = 8.9741 (12) Å	θ = 1.8–25.0°
*c* = 11.8929 (15) Å	µ = 1.60 mm^−^^1^
α = 106.841 (2)°	*T* = 298 K
β = 102.198 (1)°	Prism, dark blue
γ = 92.897 (1)°	0.47 × 0.41 × 0.30 mm
*V* = 782.3 (2) Å^3^	

### Data collection

**Table:**

Rigaku SCXmini diffractometer	2714 independent reflections
Radiation source: Rotating Anode	2280 reflections with *I* > 2σ(*I*)
graphite	*R*_int_ = 0.016
Detector resolution: 8.192 pixels mm^-1^	θ_max_ = 25.0°, θ_min_ = 1.8°
ω scans	*h* = −9→9
Absorption correction: multi-scan (*CrystalClear*; Rigaku, 2005)	*k* = −8→10
*T*_min_ = 0.520, *T*_max_ = 0.645	*l* = −13→14
4114 measured reflections	

### Refinement

**Table:**

Refinement on *F*^2^	Secondary atom site location: difference Fourier map
Least-squares matrix: full	Hydrogen site location: inferred from neighbouring sites
*R*[*F*^2^ > 2σ(*F*^2^)] = 0.030	H-atom parameters constrained
*wR*(*F*^2^) = 0.074	*w* = 1/[σ^2^(*F*_o_^2^) + (0.0271*P*)^2^ + 0.5072*P*] where *P* = (*F*_o_^2^ + 2*F*_c_^2^)/3
*S* = 1.06	(Δ/σ)_max_ = 0.001
2714 reflections	Δρ_max_ = 0.40 e Å^−^^3^
218 parameters	Δρ_min_ = −0.39 e Å^−^^3^
1 restraint	Extinction correction: *SHELXL97* (Sheldrick, 2008), Fc^*^=kFc[1+0.001xFc^2^λ^3^/sin(2θ)]^-1/4^
Primary atom site location: structure-invariant direct methods	Extinction coefficient: 0.0320 (19)

### Special details

**Table:**

Geometry. All e.s.d.'s (except the e.s.d. in the dihedral angle between two l.s. planes) are estimated using the full covariance matrix. The cell e.s.d.'s are taken into account individually in the estimation of e.s.d.'s in distances, angles and torsion angles; correlations between e.s.d.'s in cell parameters are only used when they are defined by crystal symmetry. An approximate (isotropic) treatment of cell e.s.d.'s is used for estimating e.s.d.'s involving l.s. planes.
Refinement. Refinement of *F*^2^ against ALL reflections. The weighted *R*-factor *wR* and goodness of fit *S* are based on *F*^2^, conventional *R*-factors *R* are based on *F*, with *F* set to zero for negative *F*^2^. The threshold expression of *F*^2^ > σ(*F*^2^) is used only for calculating *R*-factors(gt) *etc*. and is not relevant to the choice of reflections for refinement. *R*-factors based on *F*^2^ are statistically about twice as large as those based on *F*, and *R*- factors based on ALL data will be even larger.

### Fractional atomic coordinates and isotropic or equivalent isotropic displacement parameters (Å^2^)

**Table:**

	*x*	*y*	*z*	*U*_iso_*/*U*_eq_	
Cu1	0.19716 (5)	0.40689 (4)	0.59865 (3)	0.03271 (17)	
Cl1	0.01878 (15)	0.28462 (12)	−0.04586 (8)	0.0556 (3)	
N1	0.2799 (3)	0.5546 (3)	0.5259 (2)	0.0322 (6)	
N2	0.3181 (3)	0.5696 (3)	0.7513 (2)	0.0329 (6)	
N3	0.4694 (4)	0.1517 (4)	0.6974 (3)	0.0448 (7)	
O1	0.0888 (3)	0.2574 (2)	0.44856 (19)	0.0391 (6)	
O2	0.0886 (3)	0.2783 (3)	0.6801 (2)	0.0380 (6)	
H2A	0.1681	0.2270	0.7049	0.046*	
H2B	0.0040	0.2104	0.6350	0.046*	
O3	0.4553 (4)	0.2453 (3)	0.6385 (3)	0.0632 (8)	
O4	0.5942 (5)	0.0744 (5)	0.6999 (4)	0.0921 (12)	
O5	0.3572 (3)	0.1310 (3)	0.7528 (2)	0.0513 (7)	
O6	0.8275 (3)	0.0554 (3)	0.5520 (2)	0.0496 (7)	
H6A	0.8662	−0.0326	0.5462	0.059*	
H6B	0.7557	0.0650	0.5970	0.059*	
C1	0.2497 (4)	0.5376 (4)	0.4119 (3)	0.0322 (7)	
H1	0.2942	0.6191	0.3888	0.039*	
C2	0.1531 (4)	0.4032 (4)	0.3176 (3)	0.0304 (7)	
C3	0.0765 (4)	0.2710 (4)	0.3398 (3)	0.0323 (7)	
C4	−0.0198 (5)	0.1487 (4)	0.2394 (3)	0.0381 (8)	
H4	−0.0726	0.0618	0.2520	0.046*	
C5	−0.0380 (5)	0.1543 (4)	0.1237 (3)	0.0395 (8)	
H5	−0.1028	0.0722	0.0589	0.047*	
C6	0.0411 (5)	0.2835 (4)	0.1032 (3)	0.0375 (8)	
C7	0.1338 (4)	0.4054 (4)	0.1972 (3)	0.0377 (8)	
H7	0.1850	0.4911	0.1822	0.045*	
C8	0.3778 (5)	0.7018 (4)	0.6111 (3)	0.0382 (8)	
H8A	0.3138	0.7891	0.6030	0.046*	
H8B	0.4901	0.7184	0.5928	0.046*	
C9	0.4047 (4)	0.6945 (4)	0.7380 (3)	0.0328 (7)	
C10	0.5091 (5)	0.8088 (4)	0.8357 (3)	0.0420 (9)	
H10	0.5697	0.8927	0.8243	0.050*	
C11	0.5229 (5)	0.7975 (4)	0.9502 (3)	0.0455 (9)	
H11	0.5938	0.8729	1.0171	0.055*	
C12	0.4294 (5)	0.6720 (4)	0.9639 (3)	0.0458 (9)	
H12	0.4340	0.6634	1.0405	0.055*	
C13	0.3297 (5)	0.5603 (4)	0.8633 (3)	0.0417 (9)	
H13	0.2682	0.4755	0.8730	0.050*	

### Atomic displacement parameters (Å^2^)

**Table:**

	*U*^11^	*U*^22^	*U*^33^	*U*^12^	*U*^13^	*U*^23^
Cu1	0.0408 (3)	0.0262 (2)	0.0313 (2)	−0.00137 (16)	0.00910 (17)	0.00954 (17)
Cl1	0.0840 (8)	0.0510 (6)	0.0315 (5)	0.0041 (5)	0.0125 (5)	0.0137 (4)
N1	0.0379 (16)	0.0245 (14)	0.0342 (15)	−0.0011 (11)	0.0080 (12)	0.0102 (12)
N2	0.0406 (16)	0.0255 (14)	0.0325 (15)	0.0042 (12)	0.0070 (12)	0.0101 (12)
N3	0.0422 (19)	0.0434 (18)	0.0477 (19)	−0.0011 (15)	0.0108 (15)	0.0134 (15)
O1	0.0565 (15)	0.0288 (12)	0.0320 (13)	−0.0077 (11)	0.0110 (11)	0.0112 (10)
O2	0.0428 (14)	0.0348 (13)	0.0371 (13)	−0.0023 (10)	0.0100 (10)	0.0131 (11)
O3	0.0651 (19)	0.0525 (17)	0.093 (2)	0.0078 (14)	0.0379 (17)	0.0412 (17)
O4	0.071 (2)	0.121 (3)	0.132 (3)	0.050 (2)	0.055 (2)	0.083 (3)
O5	0.0485 (16)	0.0679 (18)	0.0474 (15)	0.0065 (13)	0.0170 (13)	0.0288 (14)
O6	0.0546 (16)	0.0384 (14)	0.0572 (16)	−0.0014 (12)	0.0160 (13)	0.0162 (13)
C1	0.0331 (18)	0.0297 (17)	0.0395 (19)	0.0029 (14)	0.0115 (14)	0.0174 (15)
C2	0.0323 (17)	0.0266 (16)	0.0342 (17)	0.0037 (13)	0.0081 (14)	0.0119 (14)
C3	0.0364 (18)	0.0289 (17)	0.0338 (18)	0.0060 (14)	0.0105 (14)	0.0111 (14)
C4	0.046 (2)	0.0278 (18)	0.0387 (19)	−0.0032 (15)	0.0114 (16)	0.0083 (15)
C5	0.044 (2)	0.0333 (19)	0.0356 (19)	0.0023 (16)	0.0081 (15)	0.0043 (15)
C6	0.045 (2)	0.0371 (19)	0.0306 (18)	0.0078 (16)	0.0102 (15)	0.0099 (16)
C7	0.042 (2)	0.0374 (19)	0.0407 (19)	0.0049 (16)	0.0134 (15)	0.0194 (16)
C8	0.045 (2)	0.0274 (17)	0.0402 (19)	−0.0051 (15)	0.0077 (16)	0.0113 (15)
C9	0.0339 (18)	0.0261 (17)	0.0377 (18)	0.0064 (14)	0.0072 (14)	0.0091 (15)
C10	0.043 (2)	0.0322 (19)	0.046 (2)	−0.0008 (16)	0.0046 (16)	0.0095 (17)
C11	0.051 (2)	0.037 (2)	0.038 (2)	0.0044 (17)	−0.0016 (17)	0.0046 (17)
C12	0.062 (3)	0.039 (2)	0.0327 (19)	0.0069 (18)	0.0041 (17)	0.0095 (17)
C13	0.054 (2)	0.0351 (19)	0.0377 (19)	0.0024 (17)	0.0105 (17)	0.0140 (16)

### Geometric parameters (Å, °)

**Table:**

Cu1—O1	1.889 (2)	C2—C3	1.421 (4)
Cu1—N1	1.936 (3)	C3—C4	1.407 (4)
Cu1—O2	1.975 (2)	C4—C5	1.368 (5)
Cu1—N2	1.982 (3)	C4—H4	0.9300
Cl1—C6	1.747 (3)	C5—C6	1.396 (5)
N1—C1	1.288 (4)	C5—H5	0.9300
N1—C8	1.469 (4)	C6—C7	1.359 (5)
N2—C13	1.343 (4)	C7—H7	0.9300
N2—C9	1.349 (4)	C8—C9	1.500 (4)
N3—O4	1.233 (4)	C8—H8A	0.9700
N3—O3	1.236 (4)	C8—H8B	0.9700
N3—O5	1.247 (4)	C9—C10	1.379 (4)
O1—C3	1.318 (4)	C10—C11	1.376 (5)
O2—H2A	0.8500	C10—H10	0.9300
O2—H2B	0.8500	C11—C12	1.383 (5)
O6—H6A	0.8500	C11—H11	0.9300
O6—H6B	0.8499	C12—C13	1.372 (5)
C1—C2	1.433 (4)	C12—H12	0.9300
C1—H1	0.9300	C13—H13	0.9300
C2—C7	1.414 (4)		
			
O1—Cu1—N1	93.94 (10)	C3—C4—H4	119.1
O1—Cu1—O2	88.85 (9)	C4—C5—C6	119.9 (3)
N1—Cu1—O2	171.60 (10)	C4—C5—H5	120.1
O1—Cu1—N2	176.81 (10)	C6—C5—H5	120.1
N1—Cu1—N2	82.98 (11)	C7—C6—C5	120.6 (3)
O2—Cu1—N2	94.32 (10)	C7—C6—Cl1	120.8 (3)
C1—N1—C8	118.5 (3)	C5—C6—Cl1	118.5 (3)
C1—N1—Cu1	125.9 (2)	C6—C7—C2	120.6 (3)
C8—N1—Cu1	115.6 (2)	C6—C7—H7	119.7
C13—N2—C9	118.7 (3)	C2—C7—H7	119.7
C13—N2—Cu1	125.8 (2)	N1—C8—C9	109.7 (3)
C9—N2—Cu1	115.3 (2)	N1—C8—H8A	109.7
O4—N3—O3	120.0 (3)	C9—C8—H8A	109.7
O4—N3—O5	118.9 (3)	N1—C8—H8B	109.7
O3—N3—O5	121.1 (3)	C9—C8—H8B	109.7
C3—O1—Cu1	127.6 (2)	H8A—C8—H8B	108.2
Cu1—O2—H2A	105.5	N2—C9—C10	121.7 (3)
Cu1—O2—H2B	115.4	N2—C9—C8	115.8 (3)
H2A—O2—H2B	106.1	C10—C9—C8	122.5 (3)
H6A—O6—H6B	107.8	C11—C10—C9	119.5 (3)
N1—C1—C2	125.3 (3)	C11—C10—H10	120.3
N1—C1—H1	117.3	C9—C10—H10	120.3
C2—C1—H1	117.3	C10—C11—C12	118.7 (3)
C7—C2—C3	119.4 (3)	C10—C11—H11	120.6
C7—C2—C1	117.3 (3)	C12—C11—H11	120.6
C3—C2—C1	123.4 (3)	C13—C12—C11	119.4 (3)
O1—C3—C4	118.5 (3)	C13—C12—H12	120.3
O1—C3—C2	123.8 (3)	C11—C12—H12	120.3
C4—C3—C2	117.7 (3)	N2—C13—C12	122.1 (3)
C5—C4—C3	121.8 (3)	N2—C13—H13	119.0
C5—C4—H4	119.1	C12—C13—H13	119.0
			
O1—Cu1—N1—C1	2.8 (3)	O1—C3—C4—C5	−179.8 (3)
O2—Cu1—N1—C1	−106.3 (7)	C2—C3—C4—C5	1.0 (5)
N2—Cu1—N1—C1	−178.0 (3)	C3—C4—C5—C6	0.3 (5)
O1—Cu1—N1—C8	179.4 (2)	C4—C5—C6—C7	−1.1 (5)
O2—Cu1—N1—C8	70.3 (8)	C4—C5—C6—Cl1	178.7 (3)
N2—Cu1—N1—C8	−1.4 (2)	C5—C6—C7—C2	0.5 (5)
O1—Cu1—N2—C13	−163.3 (19)	Cl1—C6—C7—C2	−179.3 (3)
N1—Cu1—N2—C13	−178.8 (3)	C3—C2—C7—C6	0.8 (5)
O2—Cu1—N2—C13	9.1 (3)	C1—C2—C7—C6	−178.7 (3)
O1—Cu1—N2—C9	12 (2)	C1—N1—C8—C9	−177.7 (3)
N1—Cu1—N2—C9	−3.5 (2)	Cu1—N1—C8—C9	5.5 (4)
O2—Cu1—N2—C9	−175.5 (2)	C13—N2—C9—C10	2.4 (5)
N1—Cu1—O1—C3	−3.4 (3)	Cu1—N2—C9—C10	−173.3 (3)
O2—Cu1—O1—C3	168.7 (3)	C13—N2—C9—C8	−176.7 (3)
N2—Cu1—O1—C3	−19 (2)	Cu1—N2—C9—C8	7.6 (4)
C8—N1—C1—C2	−178.5 (3)	N1—C8—C9—N2	−8.4 (4)
Cu1—N1—C1—C2	−2.0 (5)	N1—C8—C9—C10	172.5 (3)
N1—C1—C2—C7	−179.8 (3)	N2—C9—C10—C11	−1.4 (5)
N1—C1—C2—C3	0.7 (5)	C8—C9—C10—C11	177.7 (3)
Cu1—O1—C3—C4	−176.1 (2)	C9—C10—C11—C12	−0.8 (5)
Cu1—O1—C3—C2	3.1 (5)	C10—C11—C12—C13	1.8 (6)
C7—C2—C3—O1	179.3 (3)	C9—N2—C13—C12	−1.3 (5)
C1—C2—C3—O1	−1.2 (5)	Cu1—N2—C13—C12	173.9 (3)
C7—C2—C3—C4	−1.5 (5)	C11—C12—C13—N2	−0.8 (6)
C1—C2—C3—C4	178.0 (3)		

### Hydrogen-bond geometry (Å, °)

**Table:**

*D*—H···*A*	*D*—H	H···*A*	*D*···*A*	*D*—H···*A*
O2—H2a···O5	0.85	1.829	2.676 (4)	173.17
O2—H2a···N3	0.85	2.517	3.253 (4)	145.46
O2—H2a···O3	0.85	2.57	3.052 (4)	117.12
O2—H2b···O6^i^	0.85	1.811	2.657 (4)	173.63
O6—H6a···O1^ii^	0.85	2.083	2.915 (3)	165.91
O6—H6b···O4	0.85	1.934	2.782 (5)	176.61

Symmetry codes:  (i) *x*−1, *y*, *z*; (ii) −*x*+1, −*y*, −*z*+1.
